# Parametric Mapping for TSPO PET Imaging with Spectral Analysis Impulsive Response Function

**DOI:** 10.1007/s11307-020-01575-9

**Published:** 2021-01-21

**Authors:** Mattia Veronese, Marcello Tuosto, Tiago Reis Marques, Oliver Howes, Belen Pascual, Meixiang Yu, Joseph C. Masdeu, Federico Turkheimer, Alessandra Bertoldo, Paolo Zanotti-Fregonara

**Affiliations:** 1 Department of Neuroimaging, IoPPN, King’s College London, London, UK; 2 Department of Information Engineering, Padova University, Padova, Italy; 3 Department of Psychosis Studies, IoPPN, King’s College London, London, UK; 4 MRC London Institute of Medical Sciences, Hammersmith Hospital, London, UK; 5 Institute of Clinical Sciences (ICS), Faculty of Medicine, Imperial College London, London, UK; 6 Nantz National Alzheimer Center and Houston Methodist Research Neurological Institute, and Weill Cornell Medicine, 6670 Bertner Ave, Houston, TX 77030 USA; 7 Padova Neuroscience Centre, Padova University, Padova, Italy

**Keywords:** TSPO, PET, Spectral analysis, Parametric mapping

## Abstract

**Purpose:**

The aim of this study was to investigate the use of spectral analysis (SA) for voxel-wise analysis of TSPO PET imaging studies. TSPO PET quantification is methodologically complicated by the heterogeneity of TSPO expression and its cell-dependent modulation during neuroinflammatory response. Compartmental models to account for this complexity exist, but they are unreliable at the high noise typical of voxel data. On the contrary, SA is noise-robust for parametric mapping and provides useful information about tracer kinetics with a free compartmental structure.

**Procedures:**

SA impulse response function (IRF) calculated at 90 min after tracer injection was used as main parameter of interest in 3 independent PET imaging studies to investigate its sensitivity to (1) a TSPO genetic polymorphism (rs6971) known to affect tracer binding in a cross-sectional analysis of healthy controls scanned with [11C]PBR28 PET; (2) TSPO density with [11C]PBR28 in a competitive blocking study with a TSPO blocker, XBD173; and (3) the higher affinity of a second radiotracer for TSPO, by using data from a head-to-head comparison between [11C]PBR28 and [11C]ER176 scans.

**Results:**

SA-IRF produced parametric maps of visually good quality. These were sensitive to TSPO genotype (mean relative difference between high- and mixed-affinity binders = 25 %) and TSPO availability (mean signal displacement after 90 mg oral administration of XBD173 = 39 %). Regional averages of voxel-wise IRF estimates were strongly associated with regional total distribution volume (*V*_T_) estimated with a 2-tissue compartmental model with vascular compartment (Pearson’s *r* = 0.86 ± 0.11) but less strongly with standard 2TCM-*V*_T_ (Pearson’s *r* = 0.76 ± 0.32). Finally, SA-IRF estimates for [11C]ER176 were significantly higher than [11C]PBR28 ones, consistent with the higher amount of specific binding of the former tracer.

**Conclusions:**

SA-IRF can be used for voxel-wise quantification of TSPO PET data because it generates high-quality parametric maps, it is sensitive to TSPO availability and genotype, and it accounts for the complexity of TSPO tracer kinetics with no additional assumptions.

**Supplementary Information:**

The online version contains supplementary material available at 10.1007/s11307-020-01575-9.

## Introduction

For more than 30 years, the 18-kDa translocator protein (TSPO) has been the preferred target for neuroinflammation imaging with PET [1, 2]. TSPO is a five-transmembrane domain protein localised on the outer mitochondrial membrane in different cell types including microglia, astrocytes, endothelial cells, and, in small density, even in neurons [3]. TSPO is implicated in a wide array of vital cellular functions, including steroidogenesis, mitochondrial respiration, and cellular proliferation [4, 5]. The expression of TSPO is upregulated in activated microglia and reactive astrocytes as part of the brain immune response, and, for this property, TSPO is considered a marker of glia activation [6, 7]. Despite many limitations of TSPO, including its heterogenous distribution across cells type and its complex modulation in inflammatory and non-inflammatory conditions [8], TSPO radioligands are commonly used for inflammation imaging [9].

Quantification of TSPO with PET is challenging regardless of the probe used [10]. First-generation [^11^C]PK11195 suffers by low sensitivity and a poor signal-to-noise ratio [11], while the use of second-generation TSPO tracers (e.g. [11C]PBR28, [18F]DPA74, [11C]ER176) is limited by a single nucleotide polymorphism (rs6971) in the TSPO gene, which affects their binding affinity [12]. All TSPO radiotracers can bind plasma proteins, platelets, and monocytes to various extents, and this binding can be altered in pathological conditions affecting the peripheral immune system [13, 14]. In addition, TSPO tracers bind disproportionately to the endothelium, where the TSPO protein is expressed to a high level [15]. To overcome this challenge, an alternative 2-tissue compartmental model that includes an extra compartment for endothelial binding has been validated [15] and applied to TSPO data of different psychiatric and neurological conditions [16–19]. Compartmental models are limited by the need of arterial blood input function acquired in parallel of the PET imaging. Non-invasive blood-free modelling approaches have also been tested. These methods include the application of pseudo reference anatomical regions [20, 21], supervised clustering methods using the grey matter signal of healthy controls as normative regions [22–24], and simultaneous estimation [25]. However, the uneven distribution of TSPO in the normal brain and its changes that occur in pathological states represent another challenge for the quantification of binding and affect the use of normative region approaches [26].

Quantification of TSPO PET becomes even more challenging when it is performed at voxel level. In addition to the complexities of TSPO tracer kinetics described above, PET parametric mapping is hugely penalised by high measurement noise typical of voxel time-activity curves. Regularisation techniques have been discussed in the context of different radioligands [27–29] but never systematically applied and validated for TSPO PET imaging studies. TSPO parametric mapping with standardised uptake value (SUV) and the Logan graphical method are commonly applied to describe the radioactive uptake and estimate the distribution volume (*V*_T_), respectively. However both methods are not able to account for the complexity of TSPO radiotracer tissue kinetics, and cannot isolate the specific binding of the radioligands from their non-displaceable/non-specific signal [26, 30]. Recently, spectral analysis (SA), a data-driven basis function quantification method [31], has been successfully applied for parametric mapping of [11]PBR28 PET [32]. The method provides a statistical description of tracer tissue exchanges, accounting for its kinetic heterogeneity, without any priori assumption on the type and number of compartments necessary to describe the data [33]. SA proved to be able to quantify high-quality TSPO parametric maps using impulsive response function (IRF) at individual level [32]. However, up to date, a proper quantitative validation for the use of SA for TSPO PET parametric mapping is still missing.

For this reason, we tested the SA-IRF in 3 independent PET imaging datasets, reusing existing data. *Study 1* applied SA-IRF in a group of healthy controls with the purpose to investigate the sensitivity of IRF to TSPO genetic polymorphism as well as to test its consistency with the kinetic estimates provided by compartmental modelling. In *Study 2*, we tested SA-IRF sensitivity to changes in TSPO density in a group of subjects who underwent [11C]PBR28 PET before and after the administration of a pharmacological dose of the TSPO agonist XBD173. Finally, in *Study 3*, we applied SA-IRF to a head-to-head comparison of two different TSPO PET radioligands ([11C]ER176 and [11C]PBR28), to test the generalisation of the method to radioligands with different TSPO affinities.

## Materials and Methods

### Theory: the Use of SA-IRF for PET Kinetic Modelling and Parametric Imaging

In SA the tracer activity measured by the scanner in a given volume of interest (*C*_VOI_(*t*)) at time *t* is described as the convolution product between the arterial plasma tracer radioactivity (indicated as C_p_(*t*)) and the tissue impulse response function (*IRF*).

1 $$ {C}_{\mathrm{VOI}}(t)={C}_{\mathrm{p}}(t)\otimes IRF(t) $$

**Fig. 1.**
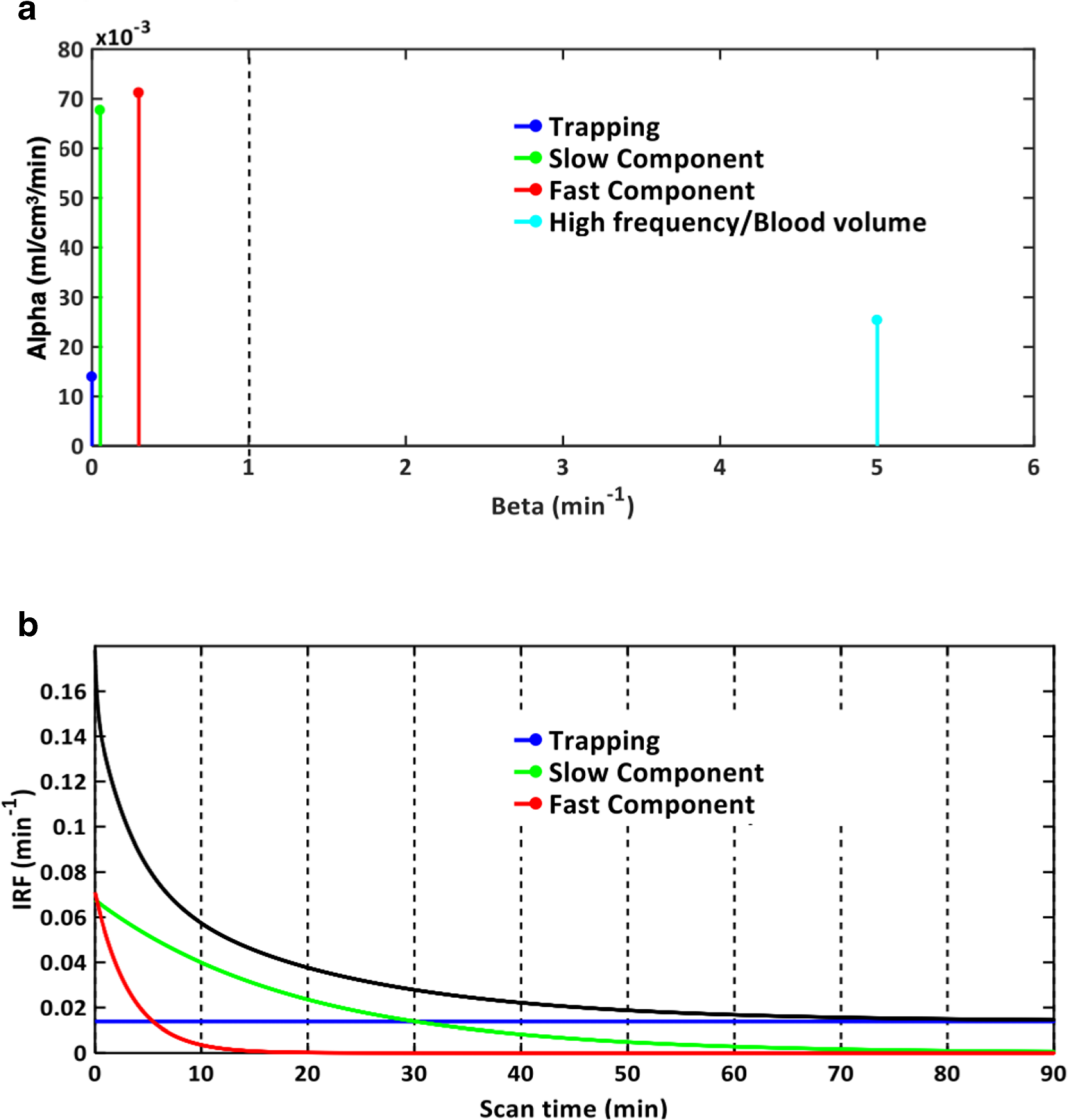
SA kinetic spectrum and impulsive response function. **a** Representative kinetic spectrum for a healthy subject, which revealed 4 different components: a trapping for *β* = 0 (blue), a slow component for *β* = 0.053 min^−1^(green), a fast component for *β* = 0.300 min^−1^ (red), and high frequency component for *β* = 5 min^−1^(cyan) likely to be associated to fractional blood volume. **b** Associated impulse response function (IRF) for tracer activity (black line) which was measured by the sum of three individual tissue components of the spectrum (blue, green, and red curves, respectively).

This description is consistent with the definition of a linear time-invariant (LTI) system, a modelling approach often used to describe biological systems in tracer kinetic experiments [34, 35]. Dynamic PET imaging is not an exception. In this formulation, C_p_(*t*) represents the input of the system and returns information on the tracer delivery to the volume of observation [36]. The *IRF*(*t*) represents the impulse response function of the system (i.e. the output of the system in case of unitary impulse input) and returns information about the tracer kinetic response in the tissues to which the tracer is delivered. Different from compartmental modelling, *IRF*(*t*) is not fixed but can be resolved as the analytical sum of *M* + 1 distinct decreasing exponential terms as:

2 $$ \mathrm{IRF}(t)=\sum \limits_{\mathrm{j}=0}^M{\alpha}_{\mathrm{j}}\cdotp {e}^{-{\beta}_{\mathrm{j}}t}\ \mathrm{with}\ {\beta}_{\mathrm{j}}\ge 0 $$

where *β*_j_ (*β*_0_ = 0, *β*_1_ < *β*_2_ < … < *β*_*M*_, unit 1/min) are the spectral components and *α*_j_ (unit, ml/cm^3^/min) the correspondent amplitudes. *M* + 1 represents the maximum number of terms to be included in the model, and this is, in general, arranged in a large set (generally between 100 and 1000). The values of *β*_*j*_ are predetermined and fixed in order to cover an appropriate spectral range from the slowest possible event of the tracer in the tissue up to a value appropriate to transient phenomena (e.g. the passage of activity through the tissue vasculature) [37]. The values of *α*_j_ are estimated from the blood and tissue time-activity curves by a non-negative least squares (NNLS) procedure. In practice, only a few components with *α*_j_ > 0 are detected, originating what is called the kinetic spectrum of the tracer in the tissues (Fig. 1).

When a particular system meets the conditions to be modelled with SA [38], the unique identifiability of some macro-parameters of interest is guaranteed, and numerical estimates can be derived directly from the kinetic spectrum [33]. These parameters include the influx rate constant (${K}_1=\sum \limits_{\mathrm{j}=1}^M{\alpha}_{\mathrm{j}}$, unit ml/cm^3^/min), the net uptake of the tracer in the tissues (*K*_i_ = *α*_0_ , unit ml/cm^3^/min), and the volume of distribution (${V}_{\mathrm{T}}=\sum \limits_{\mathrm{j}=1}^M{\alpha}_{\mathrm{j}}/{\beta}_{\mathrm{j}}$ with *β*_j_ ≠ 0, unit ml/cm^3^). The latter is the main parameter of interest used in TSPO PET studies as indirect proxy of TSPO availability and ultimately of glia activation and neuroinflammation. Alternatively, SA *IRF*(*t*) can be used directly as a statistical proxy of tracer binding [39]. In fact, by calculating IRF at a time *t*^∗^ approaching the end of the study, the relative contribution of the fast frequency components would be negligible over the slow frequency ones. Under the assumptions that high frequency components are associated to measurement noise and fast tracer kinetics (i.e. blood to tissue transport), and slow frequency components to specific binding, this would correspond to a low-pass filter removing noise and isolating the informative signal. As a consequence, *IRF*(*t*^∗^) is able to provide excellent parametric maps [32], preserving a model-free data-driven approach (Fig. 1b).

### Implementation of SA-IRF

In this study we used SA-IRF TSPO PET parametric mapping. SA-IRF estimates were derived from Eq. (2 and calculated at *t*^∗^=90 min. This time corresponds to a typical length of C-11 labelled dynamic PET imaging experiments and represents the latest available measure. The SA model was implemented using a grid of 100 components, ranging from 0.005 min^1^ to 5 min^−1^ and distributed logarithmically, consistently with other studies [40–42]. The measurement equation also included the blood volume fraction (*V*_b_, unitless) as:

3 $$ {C}_{\mathrm{measured}}(t)=\left(1-{V}_{\mathrm{b}}\right)\cdotp {C}_{\mathrm{VOI}}(t)+{V}_{\mathrm{b}}\cdotp {C}_{\mathrm{b}}(t) $$

where *C*_measured_(*t*) represents the total activity measured by the scanner within a specified volume of observation, *C*_VOI_(*t*) represents the tissue kinetic activity at either region or voxel level, and *C*_b_(*t*) the whole blood tracer activity. The spectrum was estimated using lsqnonneg command as implemented in MATLAB 2015b (MathWorks), with the data weighted by inverse of noise standard deviation of the PET measurement error. This was assumed to be additive, uncorrelated, having a Gaussian distribution with zero mean and variance equal to the decay-corrected activity divided by the length of the relative scan interval, multiplied for a scale factor *γ*. The proportionality constant *γ* was estimated *a posteriori* as described by Bertoldo et al. [43]. The method was implemented using SAKE software [44].

### Study 1: SA-IRF and TSPO Genotype

*Study 1* aimed to investigate the sensitivity of SA-IRF to TSPO genotype. The new generation TSPO tracers are affected by genetic variability of TSPO binding site induced by the rs6971 single-nucleotide polymorphism [12], and recent studies have demonstrated that tracer signal in the high-affinity binders (HAB) is 25–35 % higher compared with the mixed affinity binders (MAB) [32]. We hypothesised that SA-IRF estimates would have been significantly higher in HAB participants as compared than MAB participants irrespective of the area of the brain under investigation.

For this purpose, we considered a dataset of twenty-one healthy controls (gender: 16 males/5 females, age: 38 ± 16 years, HABs/MABs: 17/4) from a previously published study [45]. Briefly, dynamic brain PET scans were acquired over 90 min, after bolus injection of 329 ± 24 MBq of [11C]PBR28. Data were acquired with Siemens Biograph™ True Point™ PET/CT scanner (Siemens Medical Systems, Germany) and reconstructed using discrete inverse Fourier transform (DIFT [46]) in 26 frames (durations: 8 × 15 s, 3 × 1 min, 5 × 2 min, 5 × 5 min, 5 × 10 min) and the inclusion of 5-mm isotropic Gaussian smoothing. Attenuation correction was CT based. Arterial blood sampling was also acquired with a combination of automatic (first 15 min) and 12 manual samples (collected at 5, 10, 15, 20, 25, 30, 40, 50, 60, 70, 80, 90 min) to generate a time-continuous metabolite-free plasma input function. Full details on experimental protocol, tracer radiosynthesis, image acquisition, blood data analysis, and image processing are reported in the original reference [45].

SA-IRF was applied at both regional and voxel level. At regional level, SA-IRF estimates were compared with *V*_T_ estimates obtained by SA [33] and with kinetic modelling. Both standard 2-tissue compartmental model (2TCM) and 2TCM with endothelial binding (2TCM-1K) [15] were included in the analysis. For both compartmental models, the kinetic parameters were quantified with non-linear least square method using the same weights used for the SA. We expected SA-IRF estimates to be correlated with *V*_T_ estimates, with the magnitude of the correlation be dependent on the consistency between the SA kinetic spectrum and the compartmental models.

At voxel level, the quality of SA-IRF maps were qualitatively investigated, while the consistency between voxel and region SA-IRF and *V*_T_ estimates was determined with Pearson’s correlation coefficient.

### Study 2: SA-IRF Sensitivity to TSPO Availability

The aim of *Study 2* was to investigate the SA-IRF sensitivity to TSPO availability, modulated by competitive blocking. We considered an existing dataset composed of 7 patients with psychosis (gender: all males, age: 46 ± 10 years, genotype: all HABs) who underwent two [11C]PBR28 PET scans before and after the administration of XBD173, a selective TSPO agonist [47]. Experimental design and imaging methods were consistent with *Study 1* and fully described in the original references [48, 49]. All subjects received an oral dose of 90 mg dose of XBD173, aimed to reach ~ 70 % of TSPO brain occupancy [49]. Therefore, we expected SA-IRF estimates to decrease significantly following XBD173 administration. Voxel-wise SA-IRF differences between baseline and blocking estimates were statistically analysed with SPM12 (Wellcome Center, London, UK). The blocking dose of XBD173 reduces the amount of specific binding, while preserving the tracer kinetic components and thus the number of compartments/components necessary to describe the data. In addition, the parametric maps for blood volume fraction (*V*_b_) and the number of kinetic components were also considered as negative control, because no change should occur in these parameters after blocking.

### Study 3: SA-IRF Sensitivity to TSPO Tracer Affinity

The aim of *Study 3* was to investigate SA-IRF sensitivity to TSPO tracer affinity, by doing head-to-head comparison between [11C]ER176 and [11C]PBR28 brain PET scans. Both tracers are characterised by significant TSPO-specific binding, with [11C]ER176 having higher affinity and specific binding than [11C]PBR28 [50, 51]. According to these characteristics, we hypothesised [11C]ER176 SA-IRF estimates to be higher than [11C]PBR28 ones across the brain [52]. For this purpose, we re-analysed a dataset of 7 healthy volunteers (gender: 2 males/5 females, age: 68 ± 5 years, HABs/MABs: 3/4) [53]. The participants had two 90-min PET scan and metabolite-corrected arterial input function in the same day: a first one with [11C]PBR28 in the morning and a second with [11C]ER176 in the afternoon (~ 3-h difference). Injections were done over 1 min with an automated pump (743 ± 56 MBq of [11C]PBR28 and 723 ± 85 MBq of [11C]ER176), and acquisitions were performed with a Philips Gemini TF 64 PET/CT scanner. Full experimental details are reported in original reference [53].

SA was used to generate IRF parametric maps at 90 min, which were then compared with a voxel-by-voxel paired *t* test using SPM12 (Wellcome Center, London, UK).

## Results

### Study 1: SA-IRF and TSPO Genotype

As hypothesised, SA-IRF ROI estimates were sensitive to TSPO polymorphism. Across all regions, MABs SA-IRF estimates were 36 % ± 4 % smaller than the corresponding HABs ones. The largest difference was found for cingulate cortex (41 %), while whole brain had the smallest (27 %). A summary of these estimate results is reported in Table 1.

**Table 1.** [11C]PBR28 SA-IRF ROI estimates and comparison with compartmental modelling and spectral analysis VT estimates

ROI	IRF estimates (min^−1^)			Correlation with VT estimates (Pearson’s *r*)		
	HABs (Mean ± SD)	MABs (Mean ± SD)	HABs-MABs Rel Diff (Mean ± SD)	SA	2TCM1K	2TCM
Whole brain	0.144 ± 0.030	0.106 ± 0.011	37 % ± 8 %	0.91	0.92	0.93
Occipital lobe	0.156 ± 0.035	0.116 ± 0.009	34 % ± 8 %	0.86	0.91	0.92
Temporal lobe	0.141 ± 0.029	0.103 ± 0.009	37 % ± 8 %	0.90	0.93	0.92
Frontal lobe	0.150 ± 0.031	0.108 ± 0.013	38 % ± 9 %	0.89	0.89	0.91
Parietal lobe	0.151 ± 0.033	0.110 ± 0.011	37 % ± 9 %	0.89	0.90	0.93
Insular cortex	0.163 ± 0.034	0.122 ± 0.015	34 % ± 8 %	0.76	0.87	0.83
Cingulate cortex	0.158 ± 0.030	0.112 ± 0.011	41 % ± 9 %	0.88	0.89	0.92
Thalamus	0.154 ± 0.030	0.110 ± 0.014	39 % ± 9 %	0.81	0.89	0.92
Hippocampus	0.142 ± 0.030	0.104 ± 0.008	37 % ± 8 %	0.68*	*0.92*	*0.93*
Striatum	0.154 ± 0.031	0.116 ± 0.015	34 % ± 8 %	0.92	0.95	0.81
Cerebellum	0.169 ± 0.037	0.123 ± 0.016	37 % ± 9 %	0.89	0.92	0.95

*Not statistically significant

*HABS* high-affinity binders, *MABS* medium-affinity binders

SA-IRF ROI estimates were positively correlated with SA-*V*_T_ (*r =* 0.85 ± 0.07), 2TCM1K-*V*_T_ (*r =* 0.91 ± 0.02), and 2TCM-*V*_T_ (*r =* 0.91 ± 0.04) when compared across the full dataset (Supplementary Fig. 1). The weakest correlations were found for the hippocampus (*r =* 0.85 ± 0.14), but the region was also characterised by the largest number of outliers (CV *V*_T_ > 10 % or *V*_T_ > 50 ml/cm^3^) and poorer *V*_T_ estimate precision as compared to other regions. Notably, the strength of the association between SA-IRF and *V*_T_ estimates depended on TSPO genotype for 2TCM-1K (HABs *r* = 0.86, MABs *r* = 0.73, *p* = 0.03 | *z* = 2.11) but not for SA (HABs *r* = 0.77, MABs *r* = 0.75, *p* = 0.79 |*z* = 0.27). SA-IRF estimates were significantly correlated with 2TCM-*V*_T_ only in HABs (r = 0.85, *p* value < 0.01) but not in MABs (*r* = 0.16, *p* value = 0.78). This might be a consequence of the number of components returned by SA (Supplementary Fig. 2): in 89 % of the regions, the kinetic spectrum was consistent with the 2TCM-1K model (i.e. 1 trapping component and 2 reversible components), but in no region, it was consistent with the standard 2TCM (i.e. no trapping and 2 reversible components). In the remaining 11 % of ROIs, the estimated spectra were inconsistent with either model, returning a higher number of components. The number of spectral components across different regions and subjects was 3.7 ± 0.5 (mean ± sd).

**Fig. 2.**
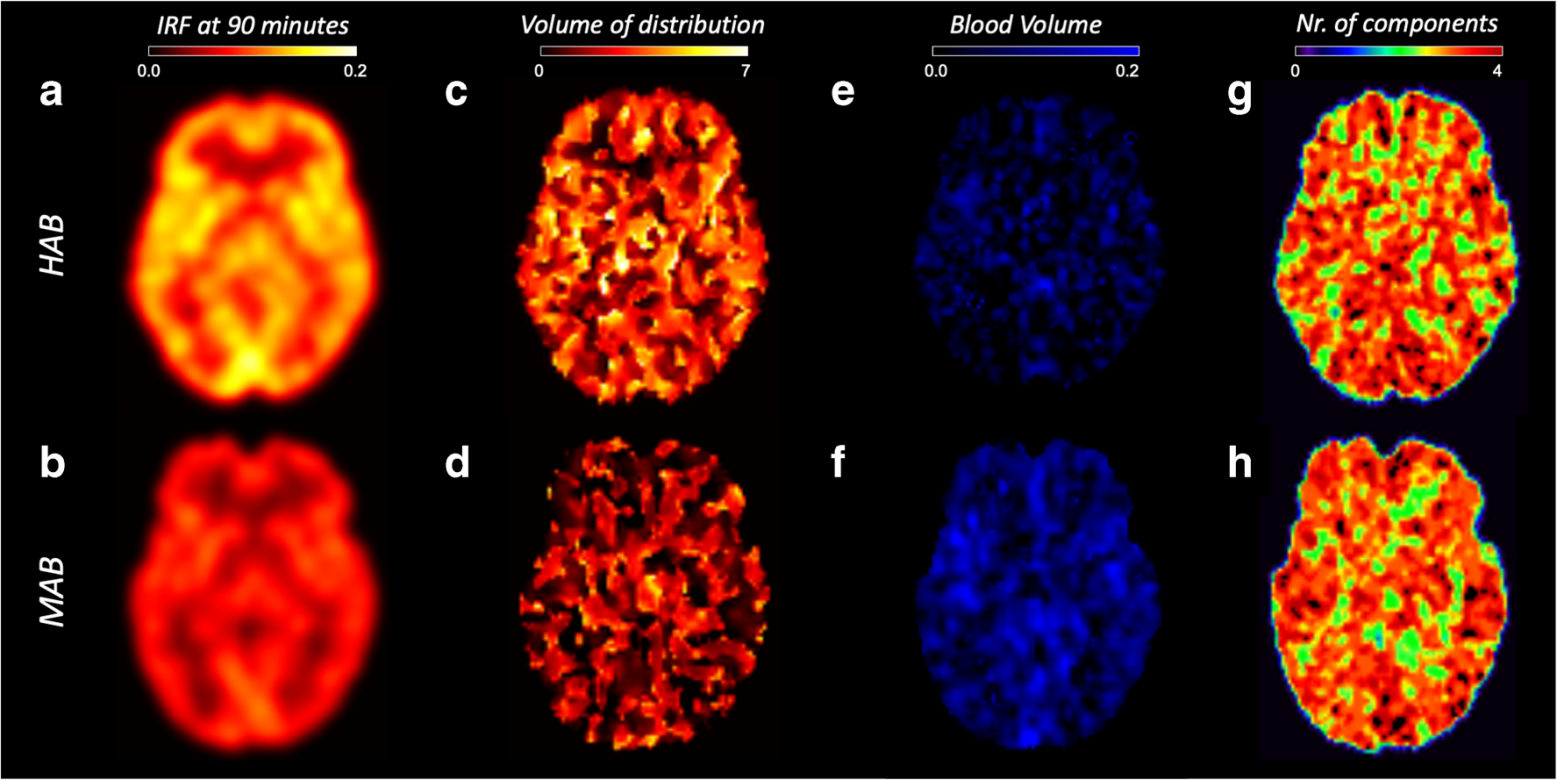
SA-IRF parametric mapping and TSPO genetic polymorphism. **a**, **b** SA-IRF (1/min). **c**, **d** SA V_*T*_ (ml/cm^*3*^). **e**, **f** SA blood volume fraction (unitless). **g**, **h** Number of components. The images show a representative HAB (top row) and MAB (bottom row) subject from a [11C]PBR28 PET imaging study. No visualisation filter is applied.

Qualitatively, SA-IRF parametric maps were spatially smooth, with regular voxel intensity within tissues of the same types (e.g. cortical regions) and grey/white matter contrast, with little noise and no outliers (i.e. negative or non-physiological estimates) (Fig. 2a, b). On the contrary, SA-*V*_T_ parametric maps were noisy and contained a significant fraction of outliers (> 20 % of the total voxels) (Fig. 2c, d). In those voxels in which SA provided reliable estimates, the kinetic spectra were consistent with the ROI analysis: 80 % of voxels matched 2TCM-1K modelling, while the remaining percentage matched the 2TCM (Fig. 2 g/h). The number of spectral components on average was 3.1 ± 0.8, irrespective of the TSPO polymorphisms.

Similar to ROI analysis, SA-IRF estimates at the voxel level were sensitive to TSPO polymorphism, with a 39 ± 2 % (mean ± SD) percentage reduction between HABs and MABs across brain regions. The largest difference was in the cerebellum and the thalamus (43 %) and the smallest in the parietal and frontal lobes (36 %). Regional averages of SA-IRF obtained from voxel estimates correlated positively (Table 2) with ROI *V*_T_ estimates, including SA-*V*_T_ (*r* = 0.86 ± 0.07), 2TCM-*V*_T_ (*r* = 0.81 ± 0.09), and 2TCM1K-*V*_T_ (*r* = 0.87 ± 0.04). In line with ROI analysis results, the correlation between SA-IRF voxel-wise regional averages and VT ROI estimates was influenced by TSPO genotype for 2TCM, and it was positively correlated in HABs (*r* = 0.73, *p* value < 0.01) but not in MABs (*r* = − 0.12, *p* value = 0.22). The correlation between the parameters was not affected by genotype for SA (HABs *r* = 0.77, MABs *r* = 0.74, *p* = 0.69 |*z* = 0.40) and 2TCM-1K (HABs *r* = 0.81, MABs *r* = 0.76, *p* = 0.44 |*z* = 0.76).

**Table 2.** [11C]PBR28 SA-IRF voxel-wise estimates and comparison with compartmental modelling and spectral analysis VT estimates

ROI	IRF estimates^#^ (min^−1^)			Correlation with VT estimates		
	HABS (Mean ± SD)	MABS (Mean ± SD)	HABs-MABs Rel Diff (Mean ± SD)	SA	2TCM-1K	2TCM
Whole brain	0.144 ± 0.030	0.104 ± 0.017	38 % ± 10 %	0.92	0.92	0.86
Occipital lobe	0.156 ± 0.037	0.111 ± 0.021	40 % ± 12 %	0.88	0.82	0.70
Temporal lobe	0.139 ± 0.030	0.100 ± 0.016	39 % ± 11 %	0.90	0.88	0.70
Frontal lobe	0.146 ± 0.031	0.107 ± 0.019	36 % ± 10 %	0.91	0.82	0.68
Parietal lobe	0.150 ± 0.034	0.110 ± 0.013	36 % ± 9 %	0.90	0.86	0.86
Insular cortex	0.166 ± 0.034	0.121 ± 0.017	37 % ± 9 %	0.74	0.83	0.84
Cingulate cortex	0.158 ± 0.030	0.112 ± 0.016	41 % ± 10 %	0.89	0.90	0.85
Thalamus	0.160 ± 0.029	0.112 ± 0.012	43 % ± 9 %	0.76	0.87	0.91
Hippocampus	0.144 ± 0.030	0.104 ± 0.012	38 % ± 9 %	0.77	0.96	0.92
Striatum	0.158 ± 0.032	0.115 ± 0.019	38 % ± 10 %	0.89	0.84	0.78
Cerebellum	0.172 ± 0.035	0.120 ± 0.028	43 % ± 13 %	0.92	0.91	0.87

^#^SA-IRF voxel-wise estimates are defined as the average of all the SA-IRF voxel estimates within each region

*HABS* high-affinity binders, *MABS* medium-affinity binders

### Study 2: SA-IRF Sensitivity to TSPO Availability

**Fig. 3.**
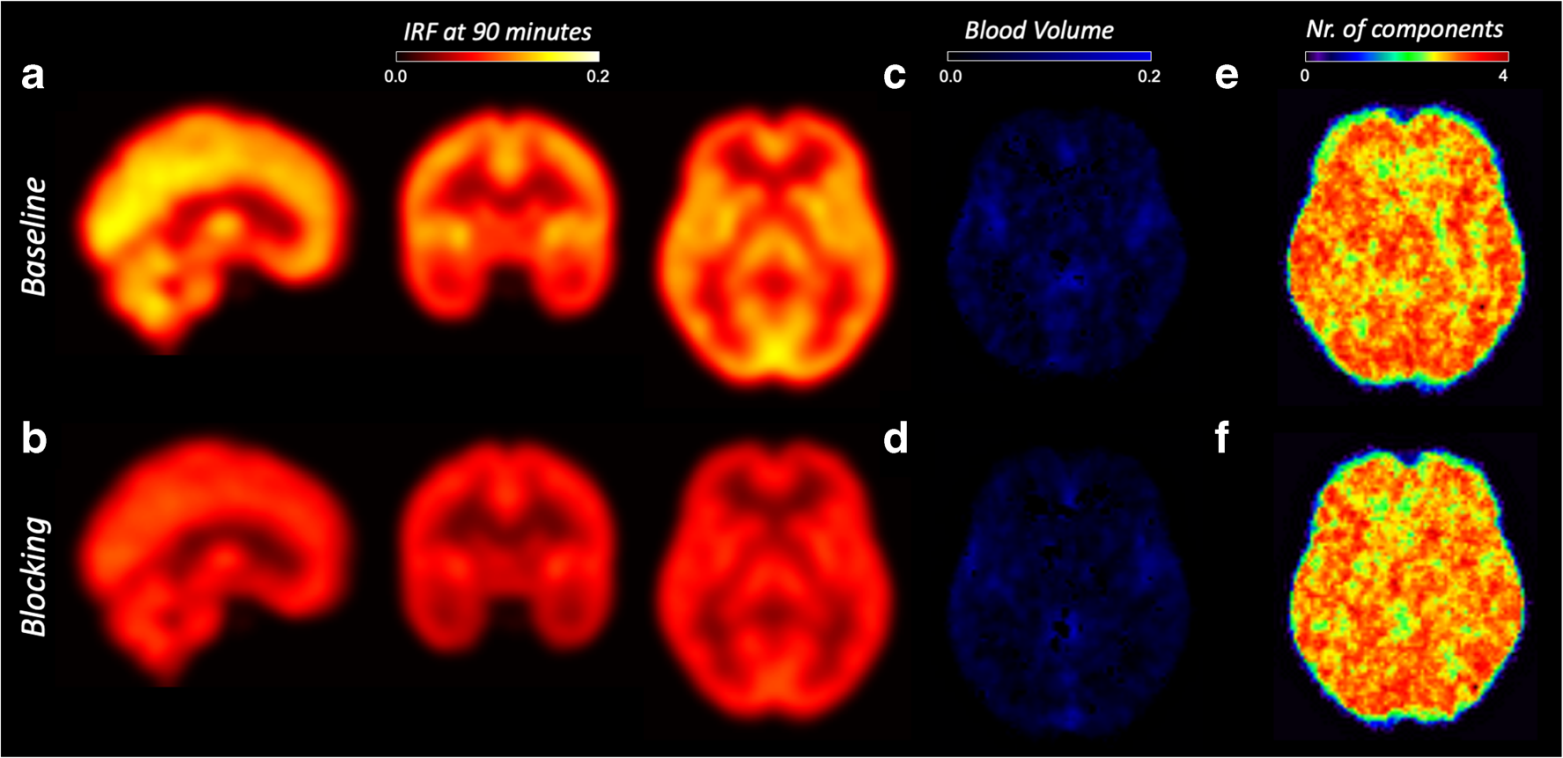
SA-IRF parametric mapping and TSPO blocking. **a**, **b** SA-IRF (1/min). **c**, **d** SA blood volume fraction (unitless). **e**, **f** Number of components. The images show a representative subject from a [11C]PBR28 PET imaging study before (top row) and after (bottom row) XBD173 blocking. No visualisation filter is applied.

Figure 3 shows the group average reduction of SA-IRF computed at 90 min before and after blocking with XBD173. The effect of blocking was statistically significant (*p* value < 0.001, no multiple comparison correction) for all the regions of interest with an average reduction of 39 ± 2 % (Table 3). The maximum displacement was in the insular cortex (ΔIRF: 41 %), and the minimum was in the thalamus (ΔIRF: 34 %). This significant displacement was confirmed by the statistical parametric analysis across all brain voxels (Supplementary Fig. 3).

**Table 3.** [11C]PBR28 SA-IRF voxel-wise estimates at baseline and after administration of blocking drug

ROI	IRF estimates (min^−1^)		
	Baseline (Mean ± SD)	Blocking (Mean ± SD)	Mean Rel Diff
Whole brain	0.106 ± 0.025	0.065 ± 0.011	39 %
Occipital lobe	0.096 ± 0.015	0.057 ± 0.009	40 %
Temporal lobe	0.090 ± 0.025	0.056 ± 0.009	38 %
Frontal lobe	0.120 ± 0.036	0.074 ± 0.013	38 %
Parietal lobe	0.101 ± 0.024	0.060 ± 0.011	40 %
Insular cortex	0.124 ± 0.036	0.072 ± 0.015	42 %
Cingulate cortex	0.129 ± 0.035	0.078 ± 0.011	39 %
Thalamus	0.121 ± 0.029	0.080 ± 0.014	34 %
Hippocampus	0.105 ± 0.024	0.064 ± 0.008	39 %
Striatum	0.117 ± 0.032	0.069 ± 0.015	41 %
Cerebellum	0.118 ± 0.028	0.071 ± 0.016	40 %

The statistical difference between the two conditions was tested and found statistically significant for each ROI (*p* < 0.05)

As hypothesised, the blocking did not alter the blood volume estimates (Vb-SA: baseline 0.03 ± 0.04, blocking 0.02 ± 0.04, *p* > 0.05) or the number of components (baseline 3.0 ± 0.76, blocking 3.0 ± 0.75, *p* > 0.05). This confirms the insensitivity of these two parameters to the TSPO availability (Fig. 3 and Supplementary Fig. 4, 5).

### Study 3: SA-IRF Sensitivity to TSPO Tracer Affinity

**Fig. 4:**
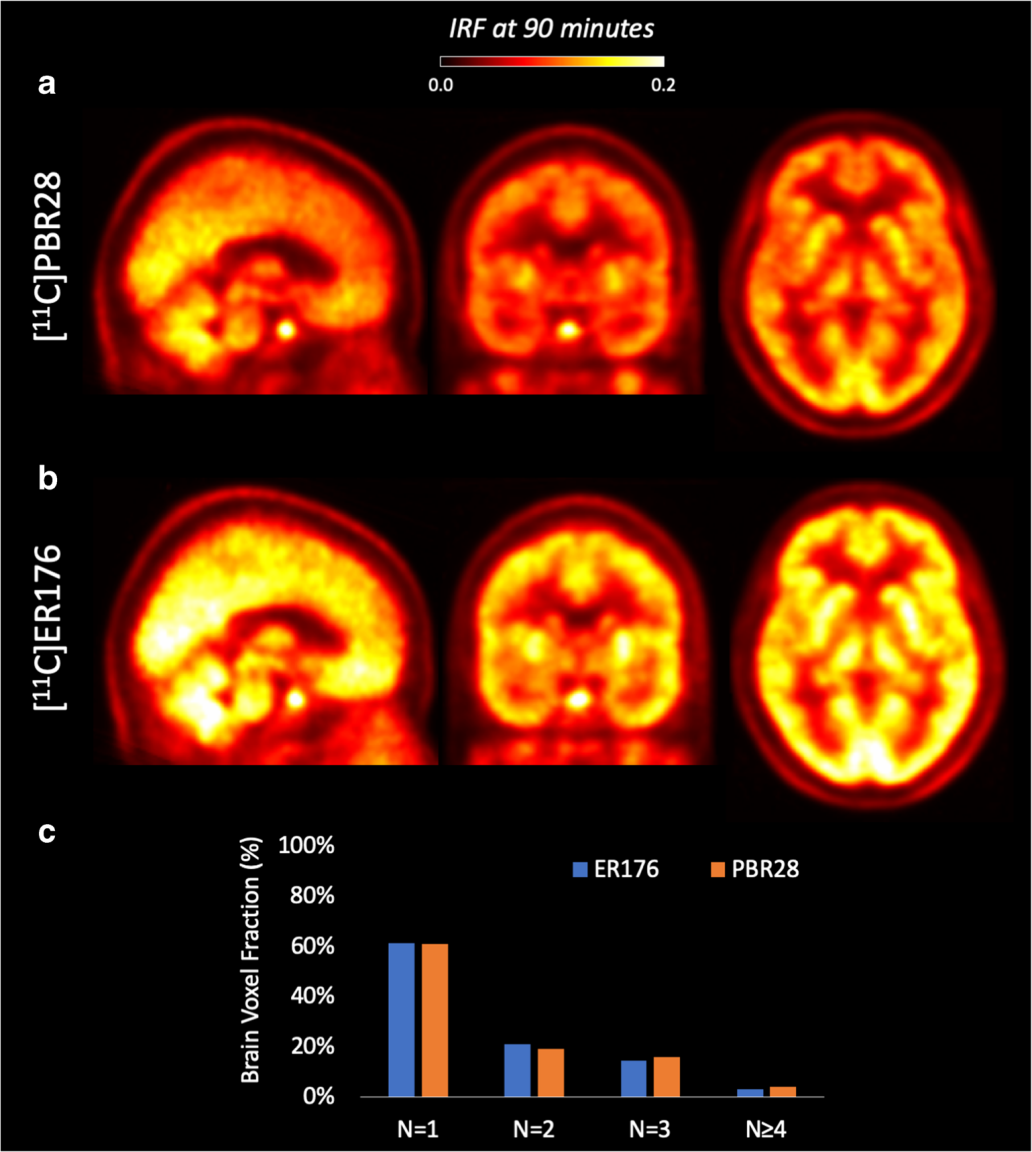
SA-IRF parametric mapping and TSPO tracer affinity. Head-to-head comparison of a [11C]PBR28 PET scan (**a**) and [11C]ER176 PET scan (**b**) for a representative healthy subject. No visualisation filter is applied. **c** Distribution of spectral components across the brain for the two radiotracers.

SA-IRF generated images of excellent quality for both TSPO PET tracers (Fig. 4). Consistently with the higher affinity and specific binding of [11C]ER176 to TSPO, IRF at 90 min was significantly higher with [11C]ER176 than with [^11^C]PRB28 across the whole brain (22 % ± 10 %). Interestingly, the distribution of spectral components did not change between the two radiotracers (Fig. 4c), indicating a similar tracer kinetics across the brain. This corroborates our previous results with Krzanowski’s tests, which showed that the covariance of the tracer distribution was the same between the two tracers [53].

## Discussion

In this work, we validated the use of SA-IRF for parametric mapping of TSPO PET imaging and demonstrated that SA-IRF (1) has the ability to differentiate between binding affinity groups due to TSPO polymorphism, (2) is sensitive to variations of TSPO concentrations, as demonstrated in baseline and blocked scans, and (3) is sensitive to the amount of specific binding, as shown by the higher values for [11C]ER176 compared to [11C]PBR28. The latter is particularly important, because it proves that SA-IRF can be extended to other TSPO PET tracers.

The use of SA-IRF as an outcome parameter has been proposed in the 90s [54] but failed to be widely adopted for PET quantification, partly because the numerical values obtained by SA-IRF are not intuitively related to the biodistribution of the radioligand. However, SA-IRF has been successfully used in several studies to quantify PET images. For instance, Hammers et al. [39] showed that the use of the IRF computed at 60 min as parameter of interest allowed the use of a wider range of bases to obtain good test–retest and reliabilities results from a database of [11C]diprenorphine studies.

Indeed, SA-IRF has several advantages. It is fast and easy to compute, and does not require any modelling assumption, since it is completely data driven. This is relevant for those tracers like TSPO that are characterised by a complex tracer kinetics in the tissues. Despite needing only few assumptions, SA-IRF is well-suited for TSPO PET imaging studies: it displays good sensitivity to genotype and to TSPO density. On the other hand, SA-IRF has some limitations. Because it is based on a statistical representation of the data, the outcome parameters are sensitive to the implementation (e.g. distribution and number of components, data binning, and time of extraction), virtually precluding the retrospective pooling of data among institutions using different experimental design protocols. On the contrary, the parameters of kinetic modelling are associated to specific biological functions and therefore remain consistent across different methodologies (e.g. a *V*_T_ estimated with 2TCM should be equal to the *V*_T_ estimated with bolus + constant infusion). As a consequence, SA-IRF is difficult to compare across different TSPO imaging studies, in absence of standardised acquisition and analysis protocol. In this respect, SA-IRF should be complementary to kinetic modelling, and not used as the only quantification approach. For the reasons described above, our database of direct head-to-head comparison between [11C]PBR28 and [11C]ER176 is particularly useful for validating SA-IRF across these two ligands. Moreover, while SA-IRF shows potential for TSPO PET imaging, studies in cross-sectional patients-*vs*-controls are needed to test the applicability of SA-IRF methodology as well its reproducibility, sensitivity, and statistical power in comparison with standard modelling approaches including Logan [55], MA1 [56], or compartmental methods solved with basis functions [57] or other regularisation strategies [27].

The presence of an additional irreversible component to model the distribution of TSPO tracers has been first postulated by Rizzo et al. [15]. Based on the histological findings of an important TSPO staining in the endothelium of arteries [23], the model hypothesises that part of the tracer is trapped in the endothelium during the transfer from the blood to the tissue. Compared with 2TCM, the 2TCM-1K model provided a more parsimonious description of the data and a better time stability correlated with mRNA transcripts of the target protein [15]. Using a database of ^18^F-DPA-714 scans, Wimberley et al. [58] subsequently showed that accounting for endothelial TSPO improved the fit of PET data and revealed a high correlation between the rate constant into the endothelial compartment and TSPO mRNA. The present study corroborates these previous findings by showing that spectral analysis of the [11C]PBR28 data, both at region and voxel level, identified irreversible/slowly reversible components in almost all the ROIs analysed. Although a correlation existed also for the fully reversible 2TCM *V*_T_, the level of correlation was affected by TSPO genotype, and, for some ROIs of MAB subjects, the correlation was not significant.

Finally, we validated the use of SA-IRF for the new TSPO tracer [11C]ER176. This tracer has favourable imaging characteristics compared to the existing ones: although it is still sensitive to TSPO polymorphism [51], its specific binding is so high that it allows imaging low-affinity binders as well [51]. A greater amount of specific binding would allow detecting group differences with increased statistical sensitivity. In addition, the logistics of PET studies would be greatly simplified, as subjects would not need to be genotyped individually before the scan. SA-IRF can be a useful tool to quantify [11C]ER176, as it correctly identified its higher signal compared to that of [11C]PBR28, which is likely due to a larger specific component [53].

## Conclusion

In summary, by using three different databases of TSPO scans, we showed that SA-IRF is well-suited for voxel-wise quantification of TSPO PET data. SA-IRF generates high-quality parametric maps, is sensitive to TSPO availability and individual TSPO genotype, and reveals information of tissue tracer kinetics comparable to compartmental modelling analysis.

## Supplementary Information

ESM 1 (PDF 333 kb)

## Data Availability

The datasets used in this study are available from the corresponding author on reasonable request.

## Abbreviations

2TCM: two-tissue compartment model
2TCM-1K: two-tissue compartment model with vascular TSPO compartment
SA: spectral analysis
IRF: impulse response function
PET: positron emission tomography
CT: computed tomography
ROI: region of interest
SUV: standardised uptake value
VT: total distribution volume
TSPO: 18kDa translocator protein
