# Supplementary material for: Parametric Mapping for TSPO PET Imaging with Spectral Analysis Impulsive Response Function
Source: Mol Imaging Biol. 2021 Jan 21;23(4):560–71. doi: 10.1007/s11307-020-01575-9 (PMC8277653; doi:10.1007/s11307-020-01575-9)

Comparison of VT regional estimates

Supplementary Figure 1

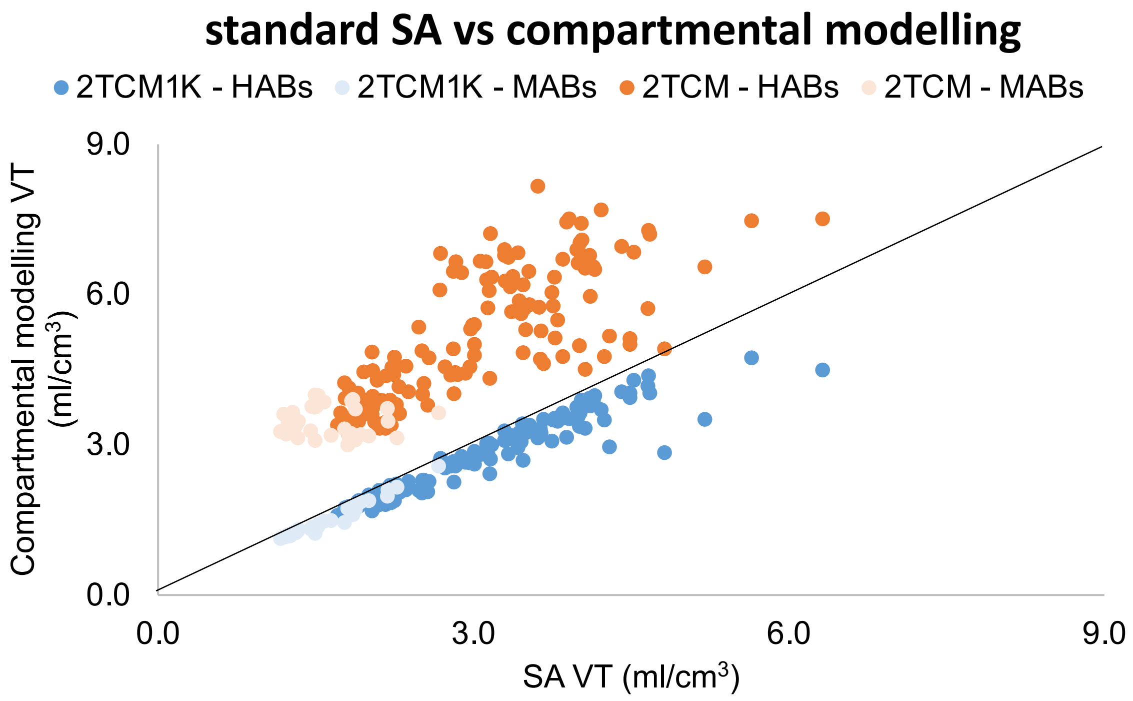

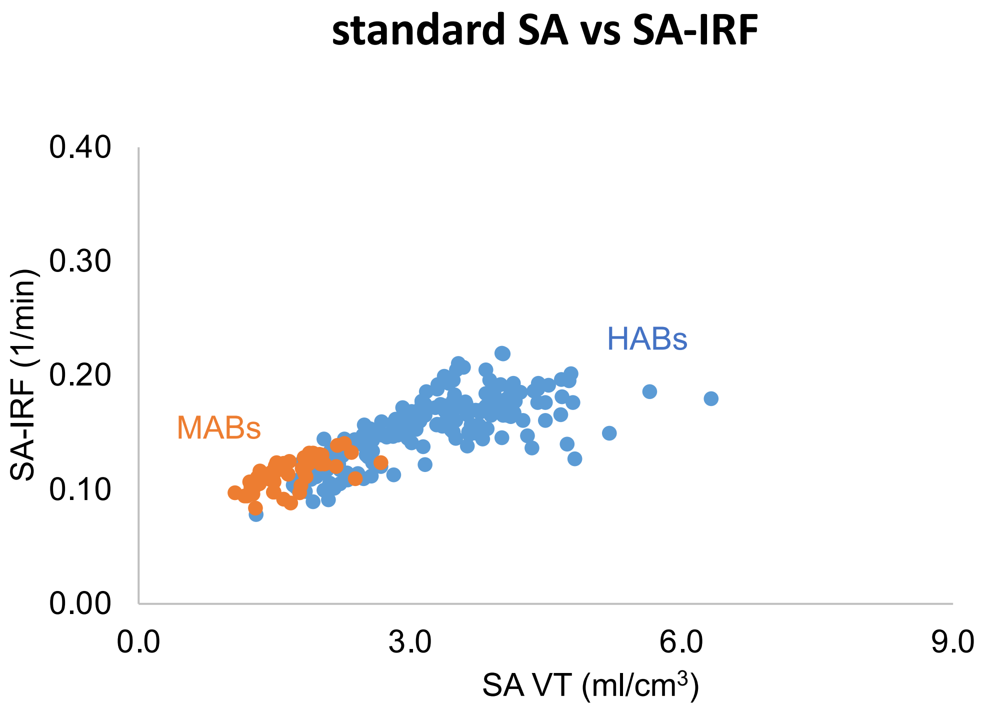

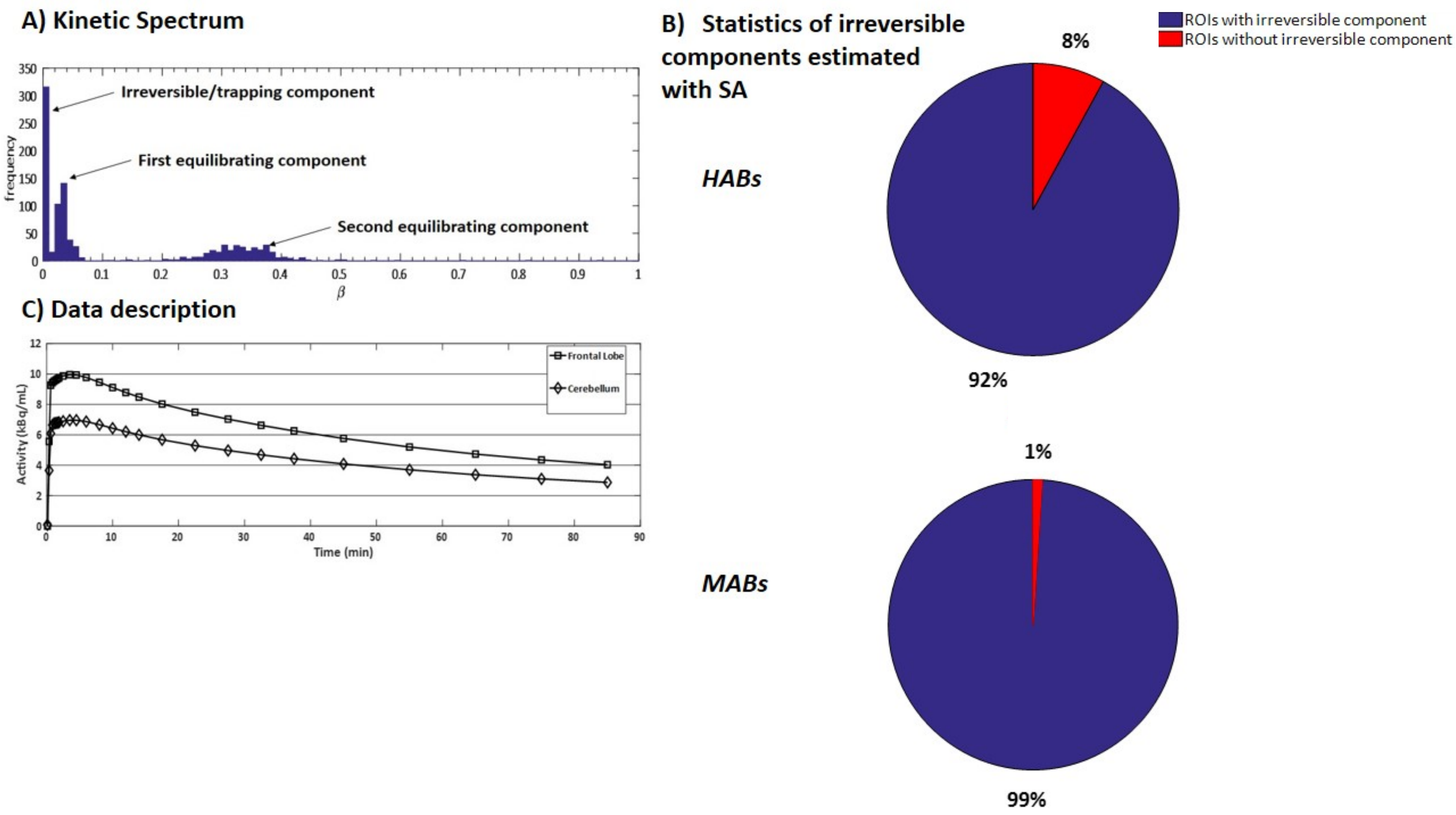
A) Kinetic Spectrum
frequency
β
Irreversible/trapping component
First equilibrating component
Second equilibrating component
B) Statistics of irreversible components estimated with SA
ROIs with irreversible component
ROIs without irreversible component
HABs
8%
92%
MABs
1%
99%
C) Data description
Activity (kBq/mL)
Time (min)
Frontal Lobe
Cerebellum

Parametric mapping for TSPO PET imaging with spectral analysis impulsive response function

Paired t-test to compare [11C]PRB28 PET SA IRF voxel estimates before and after competitive TSPO blockage with XBD173

Hypothesis: SA IRF baseline > SA IRF blocking

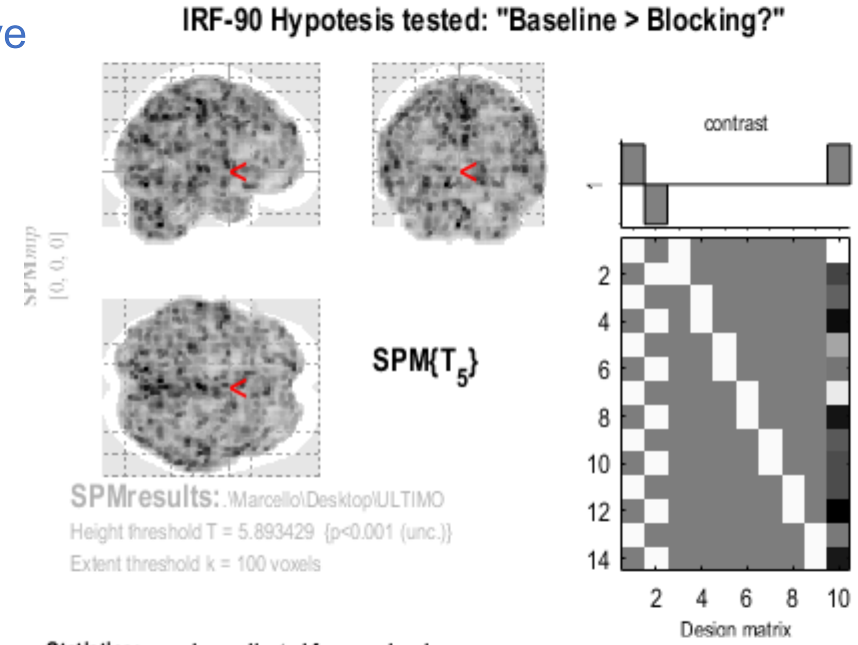

Statistics: ***p-values adjusted for search volume***

	cluster-level				peak-level					mm mm mm
	$p_{FWE\text{-}corr}$	$q_{FDR\text{-}corr}$	k_E	p_{uncorr}	$p_{FWE\text{-}corr}$	$q_{FDR\text{-}corr}$	T	$(Z_{\equiv})$	p_{uncorr}	
	0.000	0.000	96912	0.000	0.017	0.217	47.26	5.37	0.000	2 -24 50
					0.018	0.217	46.83	5.36	0.000	-2 -56 -42
					0.018	0.217	46.39	5.35	0.000	-12 -68 -12

table shows 3 local maxima more than 8.0mm apart

Height threshold: T = 5.89, p = 0.001 (1.000)
Extent threshold: k = 100 voxels, p = 0.005 (0.180)
Expected voxels per cluster, <k> = 10.680
Expected number of clusters, <c> = 0.20
FWEp: 37.957, FDRp: Inf, FWEc: 96912, FDRc: 96912

Degrees of freedom = [1.0, 5.0]
FWHM = 16.4 16.7 15.5 mm mm mm; 8.2 8.4 7.7 {voxels}
Volume: 3345448 = 418181 voxels = 747.4 resels
Voxel size: 2.0 2.0 2.0 mm mm mm; (resel = 529.80 voxels)

Parametric mapping for TSPO PET imaging with spectral analysis impulsive response function

Supplementary Figure 4

Paired t-test to compare [11C]PRB28 PET SA number of spectral components before and after competitive TSPO blockage with XBD173

Hypothesis: no difference between two conditions

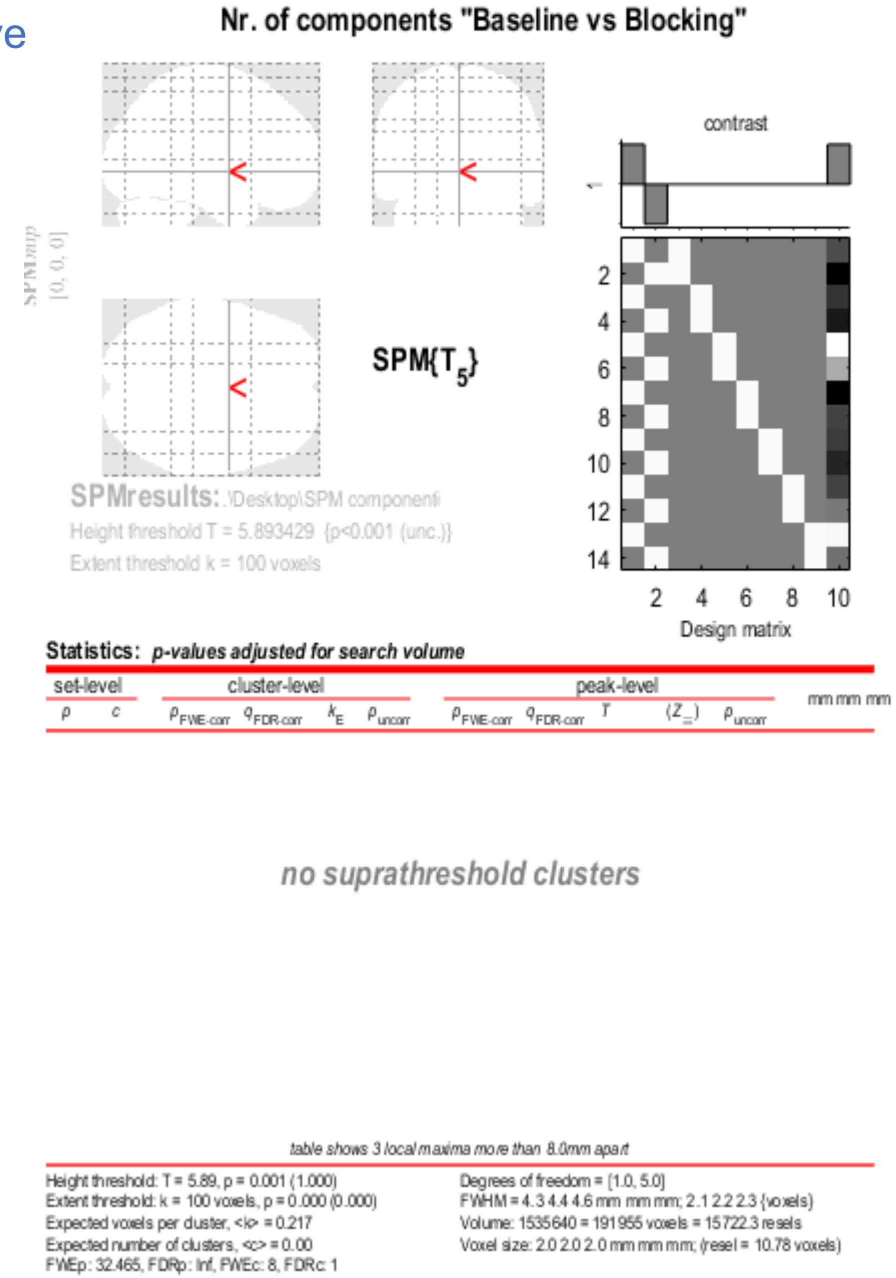

Parametric mapping for TSPO PET imaging with spectral analysis impulsive response function

Paired t-test to compare [11C]PRB28 PET blood volume before and after competitive TSPO blockage with XBD173

Hypothesis: no difference between two conditions

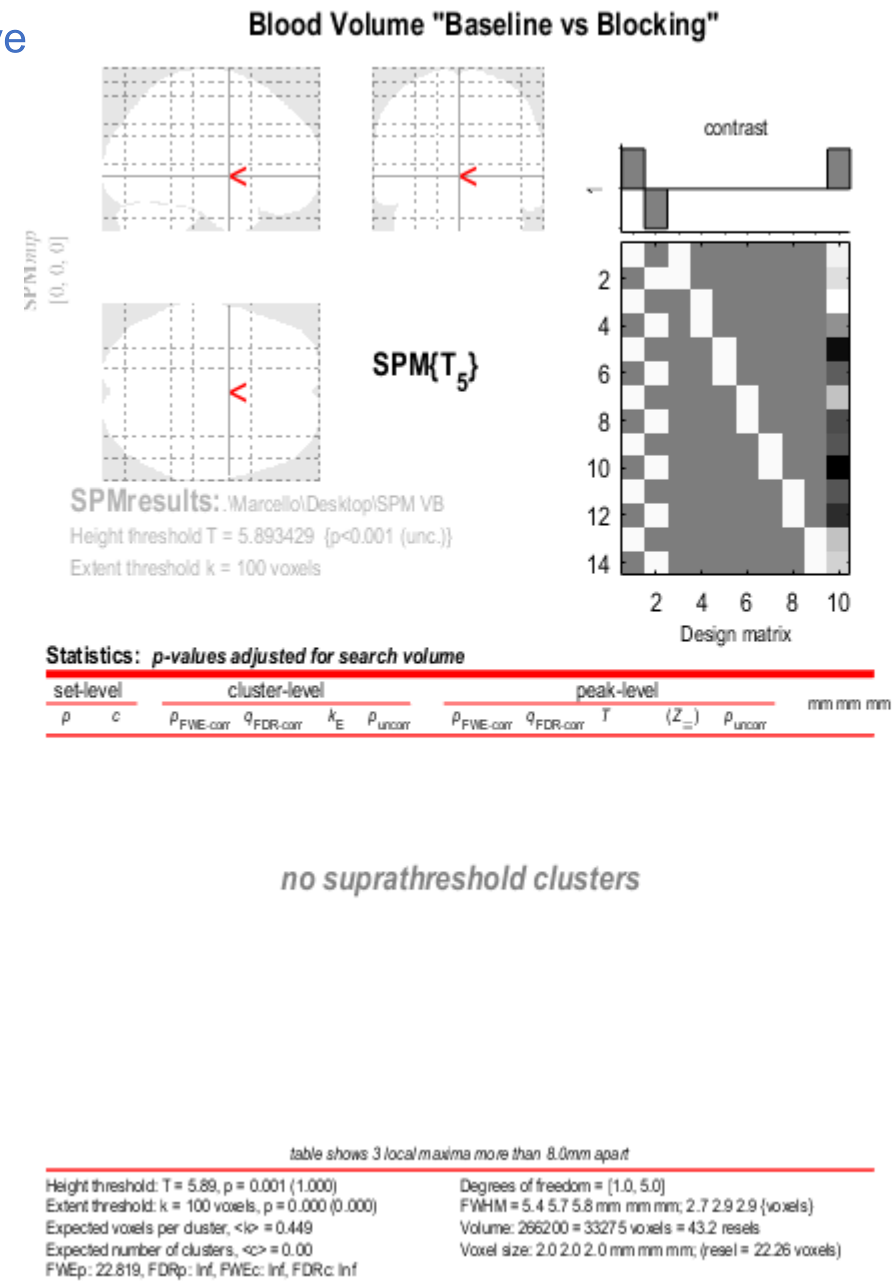

Supplement: Supplementary file 1 — (PDF 333 kb) [file 11307_2020_1575_MOESM1_ESM.pdf]
